# Cyclic-AMP Increases Nuclear Actin Monomer Which Promotes Proteasomal Degradation of RelA/p65 Leading to Anti-Inflammatory Effects

**DOI:** 10.3390/cells11091414

**Published:** 2022-04-21

**Authors:** Joseph W. Hawkins, Madeleine C. McNeill, Reza Ebrahimighaei, Harry H. Mellor, Andrew C. Newby, Mark Bond

**Affiliations:** 1 School of Translational Health Sciences, Faculty of Health Sciences, University of Bristol, Research Floor Level 7, Bristol Royal Infirmary, Bristol BS2 8HW, UK; lr18927@bristol.ac.uk (J.W.H.); madeleine.mcneill@bristol.ac.uk (M.C.M.); re15353@bristol.ac.uk (R.E.); a.newby@bris.ac.uk (A.C.N.); 2 School of Biochemistry, University of Bristol, Bristol BS8 1TD, UK; h.mellor@bristol.ac.uk

**Keywords:** Cyclic-AMP, nuclear actin, NF-κB, inflammation, RelA

## Abstract

The second messenger, cAMP has potent immunosuppressive and anti-inflammatory actions. These have been attributed, in part, to the ability of cAMP-induced signals to interfere with the function of the proinflammatory transcription factor Nuclear Factor-kappa B (NF-κB). However, the mechanisms underlying the modulation of NF-κB activity by cAMP remain unclear. Here we demonstrate an important role for cAMP-mediated increase in nuclear actin monomer levels in inhibiting NF-κB activity. Elevated cAMP or forced expression of a nuclear localised polymerisation defective actin mutant (NLS-Actin_R62D_) inhibited basal and TNFα induced mRNA levels of NF-κB-dependent genes and NF-κB-dependent reporter gene activity. Elevated cAMP or NLS-Actin_R62D_ did not affect NF-κB nuclear translocation but did reduce total cellular and nuclear RelA/p65 levels. Preventing the cAMP-induced increase in nuclear actin monomer, either by expressing a nuclear localised active mutant of the actin polymerising protein mDIA, silencing components of the nuclear actin import complex IPO9 and CFL1 or overexpressing the nuclear export complex XPO6, rescued RelA/p65 levels and NF-κB reporter gene activity in forskolin-stimulated cells. Elevated cAMP or NLS-Actin_R62D_ reduced the half-life of RelA/p65, which was reversed by the proteasome inhibitor MG132. Accordingly, forskolin stimulated association of RelA/p65 with ubiquitin affinity beads, indicating increased ubiquitination of RelA/p65 or associated proteins. Taken together, our data demonstrate a novel mechanism underlying the anti-inflammatory effects of cAMP and highlight the important role played by nuclear actin in the regulation of inflammation.

## 1. Introduction

Inflammation is an essential process for healing after tissue injury or insult. However, chronic inflammation that fails to resolve is a common, critical hallmark in the development of numerous cardiovascular diseases including atherosclerosis [1], aneurysm formation [2], vein-graft failure [3] and in stent restenosis [4]. A better understanding of how inflammatory gene expression is regulated will aid the development of novel strategies to modulate vascular inflammation, promote its resolution and treat patients suffering from the cardiovascular pathologies.

A large body of literature has characterised the role of ‘professional’ inflammatory cells, such as monocyte derived macrophages, T-lymphocytes, B-lymphocytes, neutrophils, natural killer cells and mast cells in the development of cardiovascular disease(s). However, numerous lines of evidence [5,6,7,8,9] suggest that resident vascular smooth muscle cells (VSMCs) can also adopt a pro-inflammatory phenotype that promotes vascular inflammation, disease progression and ultimately adverse clinical outcomes [10]. In normal, healthy arteries, VSMCs express a variety of contractile cytoskeletal proteins, including smooth muscle myosin heavy chain (SM-MHC), smooth muscle 22 alpha (SM22-alpha) and smooth muscle alpha actin (SM-actin) that allow them to contract and relax and regulate vascular tone and blood pressure. However, VSMCs are not terminally differentiated, retaining the ability to change their phenotype in response to a range of local environmental cues [11]. This VSMC phenotypic switch is classically defined as a switch between a contractile non-proliferative phenotype to a synthetic phenotype, associated with loss of contractile proteins and increased proliferation, migration and ECM synthesis [12]. However, VSMCs can also exhibit an inflammatory phenotype in response to stimulation with inflammatory cytokines, such as IL-1β. Importantly, the promoter regions of genes associated with this VSMC inflammatory phenotype are characterised by an over representation of binding elements for the transcription factor Nuclear Factory kappa B (NF-κB) [9]. Several lines of evidence suggest that this NF-κB-dependent inflammatory phenotype contributes towards vascular disease. For example, the activated form of NF-κB regulates the expression of many genes (including TNFα, IL-1, IL-6, VCAM1, CCL20, MMP9, MMP3 and RGS17) that are expressed in medial smooth muscle cells of early atherosclerotic lesions [13], in experimental models of atherosclerosis [14] and after vascular injury [15]. Inhibition of NF-κB blocks both early and advanced experimental atherosclerosis [16] and vascular injury-induced intima formation [17]. Clearly, VSMC can adopt a hyper-proliferative and an NF-κB-associated pro-inflammatory phenotype that contributes towards the development of vascular disease.

Nuclear factor-κB (NF-κB) consists of a family of transcription factors that play important roles in controlling inflammatory gene expression [18]. The conserved Rel-homology domain (RHD) in NF-κB family members mediates interaction with inhibitory kappa B (IκB) proteins [19], which prevent their translocation into the nucleus and consequent NF-κB-dependent gene expression [19]. Phosphorylation of IκB and its subsequent degradation represents the core regulatory mechanism of the canonical NF-κB signalling pathway [18]. Numerous signals trigger NF-κB activation, including inflammatory cytokines [18,19], oxidised LDL [20], Toll-like receptor ligands, expression of extracellular matrix (ECM)-derived products of tissue damage (DAMPs), such as fibronectin type III domain, extra domain A (EDA) [21] and changes in the composition of the extracellular matrix [22]. NF-κB activation is balanced by inhibitory signals, including stimuli that elevate intracellular levels of the second messenger 3′–5′ cyclic adenosine monophosphate (cAMP) [23]. For example, numerous reports have documented the ability of cAMP-elevating stimuli to modulate NF-κB activity in many different cell types [23,24], including in VSMC [25,26]. Although the anti-inflammatory and NF-κB-inhibitory properties of cAMP have been recognised for decades [23,27], we still do not fully understand how this is mediated. Various mechanisms have been proposed, including stimulating release of anti-inflammatory cytokines [27], increased expression of IκB [28] and post translational modification of Rel proteins [29]. However, these appear to be highly cell-type specific, possibly reflecting activation of distinct cAMP pools in response to varying upstream stimuli and/or cell-type specific responses to cAMP.

We previously demonstrated a role for actin cytoskeleton remodelling in mediating at least some (e.g., the anti-mitogenic and anti-migratory) of the effects of cAMP in VSMC [30,31,32,33,34,35]. We reported that elevated cAMP inhibits the activity of members of the Rho GTPases and inhibits cytosolic actin polymerisation [30]. This affects gene transcription, in part, by preventing nuclear localisation of transcriptional co-factors, such as MRTF-A/B [34] and YAP/TAZ [33], which control the activity of the transcription factors SRF and TEAD, respectively. Although actin is a major component of the cytoplasmic actin cytoskeleton, it has also been detected within the nucleus [36] where it has been implicated in a range of important functions, including regulation of transcription and chromatin remodelling. We recently demonstrated that elevated cAMP increases the levels of actin monomers within the nucleus and that nuclear actin dynamics are responsible for at least some of the biological effects of cAMP in VSMC [37]. However, the importance of nuclear actin in mediating the anti-inflammatory effects of cAMP is currently unknown. Here we present data demonstrating that cAMP signalling and elevated nuclear actin inhibit NF-κB activity and target gene expression by inducing the ubiquitination and proteasomal degradation of RelA/p65.

## 2. Materials and Methods

### 2.1. Reagents

All chemicals were obtained from Sigma unless otherwise stated. Antibody to RelA/p65 (#8242) was purchased from Cell Signalling Technologies. Antibody to GAPDH (MAB374) and β-actin (A1978) were purchased from Merck.

### 2.2. Smooth Muscle Cell Culture

Male Sprague Dawley rats were killed by neck dislocation in accordance with the Directive 2010/63/EU of the European Parliament and with approval from the University of Bristol ethical review board. All experiments were replicated for the number of times shown in the text and figures using different batches of cells that were prepared from different animals/donors. Human saphenous vein smooth muscle cells (HuVSMCs) were prepared as previously described [38] from spare sections of human saphenous vein obtained with informed consent in all cases from patients undergoing coronary artery bypass surgery at the Bristol Royal Infirmary. All procedures were carried out in accordance with the ethical approval (Research Ethics Committee #20/NE/0103) and the approval of the University of Bristol ethical committee. Cultures of rat aortic VSMCs (RaVSMCs) were prepared as previously described [37] and cultured in Dulbecco’s Modified Eagle’s Medium (DMEM), 1.0 g/L glucose, L-glutamine (200 mM), 10% foetal bovine serum (FBS), penicillin (100 U/mL), streptomycin (100 mg/mL).

### 2.3. Plasmids, Adenoviral Vectors and siRNA

NF-κB-dependent reporter gene plasmid, pGL4.32[luc2P/NF-κB-RE/Hygro] (abbreviated to NF-κB-LUC hereafter), containing five copies of an NF-κB response element driving transcription of a destabilised form of Firefly Luciferase was purchased from Clontech. Control luciferase reporter gene containing only a minimal promoter (minP-LUC) was described previously [33]. Expression plasmid pCMV-p65, which expresses the RelA/p65 subunit of NF-κB, was a gift from Matthew Vincenti (Dartmouth Medical School, Lebanon, NH). Plasmid pDC515:mDIA-CT expressing a constitutively active nuclear mouse mDIA was made by subcloning the F1F2+C fragment of mDIA1 from pEF-mDIA-F1F2+C plasmid (a generous gift from John Copeland and described previously [39] into the BamH1 and Sal1 sites of pDC515 (Microbix). Recombinant adenoviral vector expressing an NLS-tagged polymerisation defective mutant of β-actin (Ad:NLS-Actin_R62D_) and control adenoviral vector lacking a transgene (Ad:Control) have been described previously [37]. Plasmid expressing exportin6 (XPO6) was described previously [37]. Silencer select siRNA for IPO9 and CFL1 were purchased from Invitrogen Life Technologies.

### 2.4. Ubiquitin Affinity Assay

Affinity purification of ubiquitinated proteins was performed using the Signal-Seeker^TM^ Ubiquitination Detection Kit (Cytoskeleton, Inc., Denver, CO, USA) according to the manufacturer’s instructions. Briefly, 7 × 10^6^ cells were serum starved for 24 h before being stimulated with or without 25 µM forskolin in the presence of 10 ng/mL TNFα, 20 µg/mL cycloheximide, 20 µM MG132 for 6 h. Cells were lysed in BlastR^TM^ lysis buffer and diluted 5-fold in BlastR^TM^ dilution buffer before affinity purification of ubiquitinated proteins using Ubiquitination Affinity Beads, which are coated with ubiquitin binding domains. Affinity purified proteins were analysed by Western blotting of RelA/p65 and ubiquitin.

### 2.5. Quantitative RT-PCR and Western Blotting

Quantification of mRNA was performed using RT-qPCR, as described previously [37]. Total RNA was extracted using Ambion PureLink Total RNA extraction kits (Thermo Fisher, Waltham, MA, USA). RNA was reverse transcribed using the QuantiNova reverse transcription kit (Qiagen, Germantown, MD, USA) according to the manufacturer’s instructions. Quantitative PCR was carried out using the QuantStudio 5 Real-Time PCR instrument (Applied Biosystems, Waltham, MA, USA) with Power SYBR Green PCR Master Mix (Applied Biosystems, Waltham, MA). Primers sequences are described in Supplement Figure S1. Data were normalised to total amount of RNA. Although normalisation to either single or multiple housekeeping genes is a common approach to normalisation of qPCR data, normalisation to total RNA is also an accepted method and has previously been described as “the least problematic” [40]. Messenger RNA levels of the housekeeping gene 36B4 are presented separately from genes of interest, to demonstrate equal RNA input and quality.

### 2.6. Western Blotting

Total cell lysates were prepared in 1x reducing Laemmli sample buffer (2% SDS, 10 glycerol, 50 mM Tris pH 6.8, 2.5% β-mercaptoethanol, 0.002% bromophenol blue). Proteins were denatured by heating to 100 °C for 4 min prior to electrophoresis. Electrophoresis was performed using Bio-Rad 4–15% polyacrylamide mini-TGX gels in a Mini-Protean II system. Proteins were transferred to PVDF membrane using a semi-dry Turbo blotter system (Bio-Rad). Membranes were blocked for 1 h at room temperature in 5% low-fat milk powder in Tris buffered saline containing 0.2% Tween20 (1TBS.T) before incubation with primary antibody overnight at 4 °C. Following extensive washing in TBS.T, blots were incubated with HRP-conjugated secondary antibodies. Specific proteins were detected using Immobilon ECL reagent and a ChemiDoc-MP digital imaging system (Bio-Rad, Watford, U.K.).

### 2.7. Reporter Gene Assays and Transient Transfection

NF-κB reporter gene activity was determined by quantifying the cellular luciferase reporter activity in cells transfected with NF-κB-LUC compared to minP-LUC. Plasmid transfection was performed by electroporation of 1 × 10^6^ cells with 5 μg of plasmid DNA using an Amaxa Nucleofector-1.5 (program A-033). Cell lysates were prepared in Cell Culture Lysis reagent (Promega) and reporter gene activity measured using the Luciferase Assay System (Promega) and a GloMax Discover luminometer (Promega). For gene silencing, 1 × 10^6^ cells were transfected with 100 pmol of Silencer Select siRNA (Life Technologies) by electroporation using a Amaxa Nucleofector-1.5 (program A-033).

### 2.8. Immunocytochemistry

Using a 24-well plate, coverslips were briefly immersed in cell attachment factor (Gibco) and then allowed to dry for 20 min. Cells were trypsinised and counted as previously described before being seeded onto the coverslips at a density of 5 × 10^4^. The day after seeding, cells were stimulated as specified and fixed in 1% paraformaldehyde (PFA) for 10 min at the conclusion of stimulation. After fixation, cells were subject to 3 × 5-min washes in PBS to remove PFA and permeabilised in 0.2% Triton-X-100 diluted in PBS for 5 min. Cells were once again washed following permeabilisation in PBS. Cells were stained with Alexa-Fluore-488 conjugated NF-κB/p65 antibody (Cell Signalling Technologies; D14E12) diluted in 0.1% Triton-X-100 PBS solution overnight at 4 °C. Antibody staining was concluded with a further 3 × 5-min PBS washes. Nuclei were counter-stained with Hoechst 33342 before a final PBS rinse. To remove any excess salts, cells were rinsed with deionised water before being mounted on glass coverslips containing SlowFade Gold mounting medium. Nuclear and cytoplasmic RelA/p65 staining intensity was quantified using ImageJ software.

For quantification of nuclear actin monomer levels, cells were fixed in 4% formaldehyde for 10 min, permeabilised in 0.1% Triton-X-100 diluted in PBS for 5 min before being blocked in PBS containing 5% BSA (*w*/*v*) for 1 h. Cells were then stained with 9 ug/mL Alexa Fluor-488 conjugated DNAse1, which specifically binds actin monomer for 20 min. After washing in PBS, nuclei were stained with Hoechst 33342. Nuclear staining intensity was quantified using Cell Profiler software.

### 2.9. Cycloheximide Chase Assay

RelA/p65 protein stability was determined by time course analysis of cells treated with the protein synthesis inhibitor, cycloheximide. Serum starved cells were pretreated with forskolin (25 µM) for 1 h or infected with either Ad:Control or Ad:NLS-Actin_R62D_, 24 h before stimulation with 10 ng/mL TNFα. TNFα stimulation was performed in the presence of 20 µg/mL cycloheximide and 10 µM MG132, as indicated. Total cell lysates were analysed for RelA/p65 levels by Western blotting.

### 2.10. Statistical Analysis

Data are presented as means ± standard error. After testing data sets for normality, statistical analysis was performed using one-way ANOVA with Student-Newman-Keul’s post-test or, where appropriate, a paired Student’s *t*-test, as indicated. * Indicates *p* < 0.05, ** indicates *p* < 0.01, *** indicates *p* < 0.001.

## 3. Results

### 3.1. Nuclear Actin Repressed Genes Are Associated with Inflammatory Gene Ontology Terms and Display Enrichment of NF-κB Binding Elements in Their Promoter Regions

**Figure 1:**
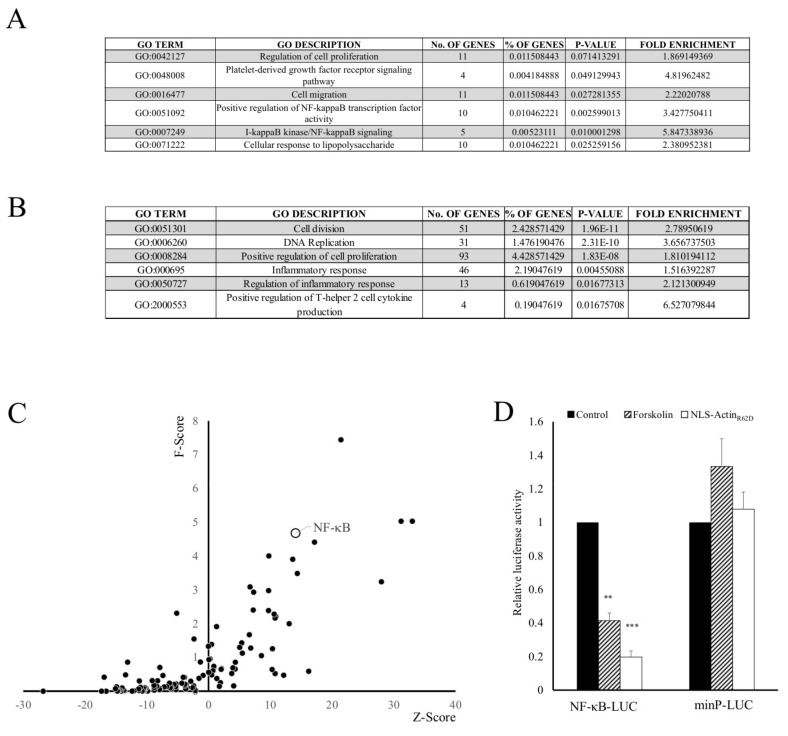
Gene Ontology and transcription factor element enrichment analysis of forskolin and nuclear actin monomer-repressed transcriptome identifies enrichment of inflammation related GO-terms and NF-κB binding elements. Gene Ontology analysis of genes identified by RNA-seq as being significantly repressed by a 6-h stimulation with 25 µM forskolin in VSMC (**A**). Gene Ontology of genes identified by RNA-seq as significantly repressed by expression of NLS-Actin_R62D_ in VSMC (**B**). Transcription factor enrichment analysis of −1000bp promoter regions of NLS-Actin_R62D_ repressed genes (**C**). VSMC were transfected with either a NF-κB-dependent luciferase reporter gene (NF-κB-LUC) or a luciferase reporter gene driven by a minimal promoter lacking NF-κB elements (minP-LUC). Luciferase activity was quantified after 6-h stimulation with 25 µM forskolin or 24 h after infection with a control adenovirus or an adenovirus expressing NLS-Actin_R62D_ (**D**). ** indicates *p* < 0.01, *** indicates *p* < 0.001.

We previously reported that elevated cAMP signalling in VSMC increases levels of nuclear actin monomer. Here we confirm this increase in nuclear actin monomer levels in VSMC after 1, 2 or 3 h following stimulation with forskolin (Supplement Figure S2). We also previously characterised the cAMP and nuclear actin monomer dependent transcriptomes in VSMC [37]. Using adenoviral-mediated expression of a nuclear targeted polymerisation defective mutant of β-actin (NLS-Actin_R62D_) and the adenylate cyclase agonist, forskolin, we identified 2425 and 1209 genes as being repressed by elevated nuclear actin or forskolin, respectively [37]. Here, we report that gene ontology (GO) enrichment analysis of forskolin repressed genes [37] demonstrates an enrichment of genes associated with the positive regulation of NF-κB transcription factor activity (GO:0051092), IκB kinase/NF-κB signalling (GO:0007249) and cellular response to lipopolysaccharide (GO:0071222) (Figure 1A), indicating regulation of genes associated with inflammation. GO analysis of nuclear actin monomer-repressed genes identified enrichment of genes associated with inflammatory response (GO:000695), regulation of inflammatory response (GO:0050727) and positive regulation of T-helper 2 cell cytokine production (GO:2000553) (Figure 1B), similarly indicating regulation of genes associated with inflammation.

Analysis of the −1000bp promoter regions of genes significantly repressed by nuclear actin monomer identified an enrichment of NF-κB binding elements (Matrix ID: MA0105.1), implying a possible causative role for NF-κB transcription factors in mediating these inflammation-related transcriptional changes. To test this hypothesis directly, we performed NF-κB-dependent reporter gene analysis to quantify the effect of elevated cAMP or nuclear actin monomer on NF-κB transcriptional activity. This demonstrated that both forskolin-mediated elevation of cAMP or increased nuclear actin monomer levels significantly inhibited NF-κB activity in serum-stimulated VSMCs, without affecting the activity of a control reporter under the control of a minimal promoter that lacks NF-κB binding elements (Figure 1D).

### 3.2. Elevated cAMP and Nuclear Actin Monomer Inhibit Serum Stimulated mRNA Levels of NF-κB Target Genes

**Figure 2:**
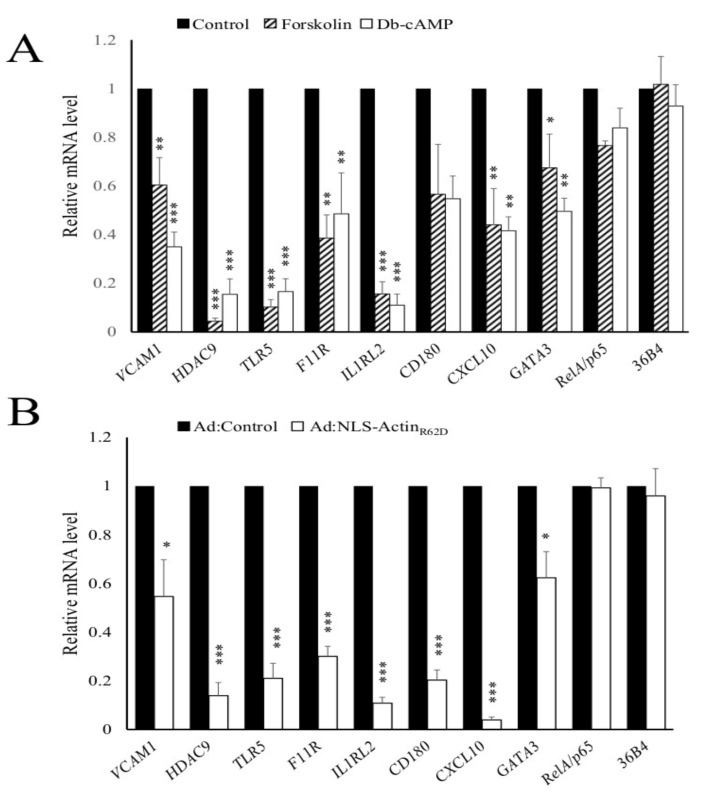
Elevated nuclear actin monomer and cAMP signalling repress basal NF-κB target gene expression in VSMC. VSMCs were stimulated with 25 µM forskolin or 200 µM Db-cAMP for 6 h. Total RNA was isolated 24 h post infection and analysed for mRNA levels of NF-κB target genes by RT-qPCR ((**A**); *n* = 5). VSMCs were infected with either Ad:Control or Ad:NLS-Actin_R62D_ (**B**). Total RNA was isolated 24 h post infection and analysed for mRNA levels of NF-κB target genes by RT-qPCR ((**B**); *n* = 4). One way ANOVA with Student Newman Keuls post test; * indicates *p* < 0.05, ** indicates *p* < 0.01, *** indicates *p* < 0.001.

From our RNA-seq data [37], we selected eight genes (VCAM1, HDAC9, TLR5, F11R, IL1RL2, CD180, CXCL10, GATA3) that were repressed by forskolin and elevated nuclear actin monomer, which also contain a consensus NF-κB binding elements in their proximal promoter regions. Cells cultured in serum were stimulated with either forskolin or the stable cAMP analogue dibutyryl-cAMP (Db-cAMP) and mRNA levels of these genes quantified using RT-qPCR. This confirmed that elevated cAMP signalling in VSMC significantly repressed mRNA expression of these NF-κB target genes (Figure 2A). To confirm that these genes are also repressed by elevated nuclear actin monomer, cells were infected with either a control adenovirus vector (Ad:Control) or an adenovirus expressing nuclear localised polymerisation defective actin mutant (Ad:NLS-Actin_R62D_). Infection with Ad:NLS-Actin_R62D_ resulted in clearly detectable expression of FLAG-tagged NLS-Actin_R62D_ protein using an anti-FLAG antibody (Supplement Figure S3). However, no increase in total cellular β-actin protein levels was detectable, indicating a small increase in nuclear actin levels relative to the highly abundant total cellular actin. Importantly, expression of NLS-Actin_R62D_ resulted in a significant repression of these NF-κB target genes (Figure 2B). Levels of NF-κB RelA/p65 mRNA were not significantly affected by elevated cAMP signalling (Figure 2A) or elevated nuclear actin monomer (Figure 2B).

### 3.3. Elevated cAMP and Nuclear Actin Monomer Repress TNFα-Stimulated Expression of NF-κB-Target Genes

**Figure 3:**
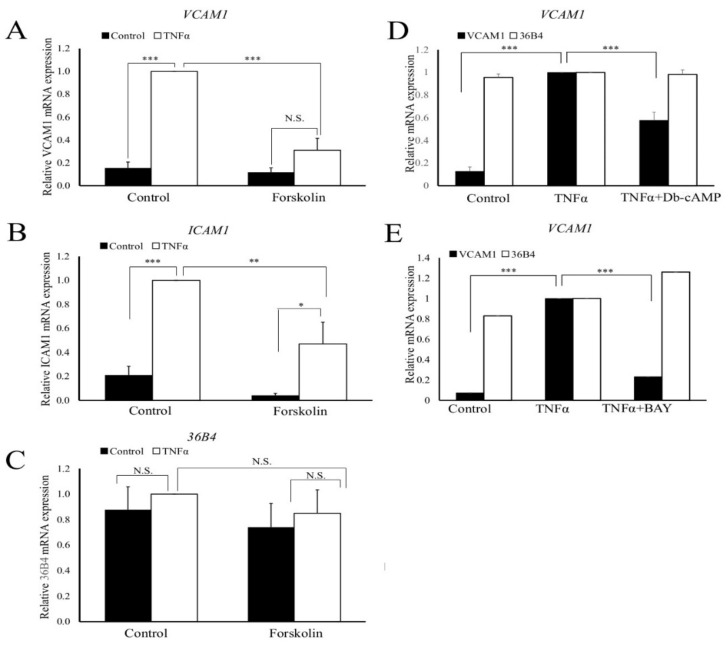
Cyclic-AMP signalling inhibits the mRNA levels of NF-κB target genes. VSMCs were serum starved for 24 h. Cells were pre-treated with 25 µM forskolin for 1 h before a 2-h stimulation with 10 ng/mL TNFα and levels of VCAM1 ((**A**); *n* = 7), ICAM1 ((**B**); *n* = 7) and 36B4 ((**C**); *n* = 7) mRNA quantified. Cells were pre-treated with 200 µM Db-cAMP for 1 h before a 2-h stimulation with 10 ng/mL TNFα and levels of VCAM1 and 36B4 mRNA quantified ((**D**); n = 5). Cells were pre-treated with 5 µg/mL BAY60-6583 for 1 h before a 2-h stimulation with 10 ng/mL TNFα and levels of VCAM1 and 36B4 mRNA quantified ((**E**); *n* = 4). One way ANOVA with Student Newman Keuls post test; * indicates *p* < 0.05, ** indicates *p* < 0.01, *** indicates *p* < 0.001. N.S.: not significantly.

We next tested if elevated cAMP and elevated nuclear actin monomer inhibits TNFα-mediated induction of classical NF-κB target genes (VCAM1 and ICAM). Cells were pre-treated with forskolin for 1 h before stimulation with TNFα for a further 2 h. As expected, TNFα stimulation resulted in a significant upregulation of VCAM1 and ICAM mRNA levels, without affecting levels of the housekeeping gene 36B4 (Figure 3A–C). Importantly, forskolin treatment significantly reduced the TNFα-mediated induction of VCAM1 mRNA levels (Figure 3A). ICAM mRNA was significantly increased by TNFα in forskolin-pre-treated cells but only to levels that were significantly lower than in cells without forskolin treatment (Figure 3B). These findings were mimicked in human VSMC, with TNFα inducing a significant increase in VCAM1 mRNA expression. This induction was significantly attenuated when cells were co-treated with forskolin (Supplement Figure S4A). In a similar manner, the synthetic cAMP analogue, Dibutyryl-cAMP (Db-cAMP; Figure 3D) and the adenosine A2B-receptor agonist, BAY65-6083 (Figure 3E) also significantly inhibited the TNFα induced mRNA levels of VCAM1, without affecting levels of the housekeeping gene, 36B4. Taken together, these data demonstrate that cAMP-signalling inhibits the mRNA expression level of TNFα-stimulated NF-κB target genes.

**Figure 4:**
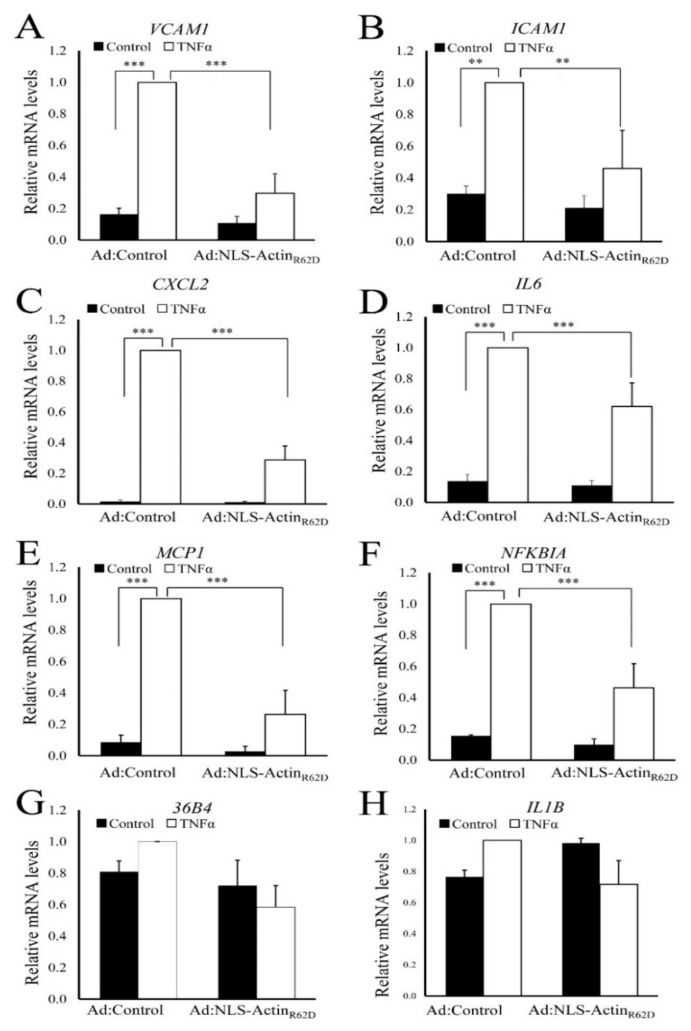
Nuclear actin monomer inhibits mRNA levels of NF-κB target genes. VSMC were infected with either control adenovirus (Ad:Control) or an adenovirus expressing NLS-Actin_R62D_ (Ad:NLS-Actin_R62D_). The next day, cells were stimulated with 10 ng/mL TNFα for 2 h. Total RNA was extracted and analysed for mRNA levels of VCAM1 (**A**), ICAM1 (**B**), CXCL2 (**C**), IL6 (**D**), MCP1 (**E**), NFKBIA (**F**), 36B4 (**G**), IL1B (**H**) using RT-qPCR. Data are *n* = 5. One way ANOVA with Student Newman Keuls post-test; ** indicates *p* < 0.01, *** indicates *p* < 0.001.

To further test the hypothesis that elevated nuclear actin monomer inhibits NF-κB-dependent gene expression, we quantified the effect of NLS-Actin_R62D_ on TNFα-stimulated mRNA levels of an expanded set of classical NF-κB target genes (VCAM1, ICAM, CXCL2, IL-6, MCP1, NFKBIA IL1B; Figure 4A–H). Expression of VCAM1, ICAM, CXCL2, IL6, MCP1 and NFKBIA but not IL1B, were all significantly increased in response to TNFα stimulation in control adenovirus (Ad:Control) infected cells (Figure 4A–H). Importantly, TNFα induction of these genes was significantly lower in cells expressing NLS-Actin_R62D._ The housekeeping gene 36B4 was not significantly altered. Note that some of the genes (*CXCL2*, *IL6*, *MCP1* and *NFKBIA*) that are repressed by nuclear actin, were not repressed by cAMP elevating stimuli (data not shown). We believe that this is due to regulation of these genes by additional transcription factors that are activated by cAMP, such as CREB.

### 3.4. Elevated cAMP and Nuclear Actin Monomer Inhibit TNFα Induced NF-κB Activity Independently of Nuclear Translocation of RelA/p65

**Figure 5:**
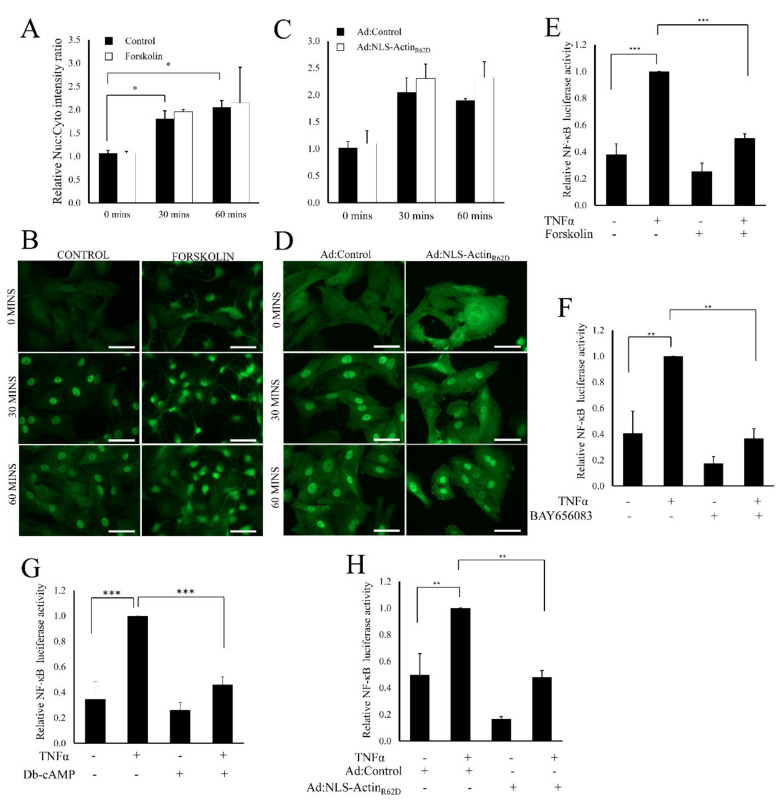
Cyclic-AMP and nuclear actin monomers attenuate TNFα-induced NF-κB activity without impairing RelA/p65 nuclear translocation. Serum starved VSMCs were pre-treated with 25 µM forskolin for 1 h prior to TNFα stimulation for the indicated times (**A**,**B**). Cells were infected with control adenovirus (Ad:Control) or virus expressing NLS-Actin_R62D_ (Ad:NLS-Actin_R62D_) 24 h before TNFα stimulation (**C**,**D**). Cytoplasmic:nuclear ratios of RelA/p65 staining intensity was quantified ((**A**,**C**); *n* = 3). Cells were transfected with the NF-κB reporter plasmid (NF-κB-LUC). Serum starved cells were treated with forskolin ((**E**); *n* = 4), BAY60-6583 ((**F**); *n* = 4) or Db-cAMP ((**G**); *n* = 4) for 1 h before TNFα stimulation for a further 6 h. Cells were transfected with NF-κB-LUC and subsequently infected with either Ad:Control or Ad:NLS-Actin_R62D_. Cells were stimulated with TNFα for 6 h before NF-κB reporter activity was quantified ((**H**); *n* = 5). ANOVA with Student Newman Keul’s post hoc test. Data are mean ± SEM. *, ** and *** indicate *p* < 0.05, 0.01 and 0.001, respectively.

To understand the mechanism by which cAMP suppresses NF-κB activity, we next tested if elevated cAMP and nuclear actin monomer inhibited TNFα induced nuclear translocation of RelA/p65 and NF-κB transcriptional activity. As expected, TNFα stimulation induced a significant increase in the nuclear:cytoplasmic ratio of RelA/p65 immunostaining after 30 and 60 min (Figure 5A–D; higher resolution images with nuclear counter stain in Supplement Figure S5). However, this ratio was not significantly different in cells pre-treated with forskolin (Figure 5A,B) or in cells expressing NLS-Actin_R62D_ (Figure 5C,D). Nevertheless, whereas TNFα stimulation also significantly increased NF-κB reporter gene activity after 6 h (Figure 5E–H), elevating cAMP levels by pre-treating cells with either with forskolin (Figure 5E), or the adenosine A2B receptor agonist BAY65-6083 (Figure 5F) or with the cyclic-AMP analogue Di-butyryl-cAMP (G; *n* = 4), significantly inhibited TNFα induced NF-κB reporter activity. Likewise, expression of NLS-Actin_R62D_ also significantly inhibited TNFα induced reporter gene activity (Figure 5H). A comparable inhibition of NF-κB reporter gene activity was reported amongst human VSMC, with forskolin (Supplement Figure S6A), BAY65-6083 (Supplement Figure S6B) and NLS-Actin_R62D_ (Supplement Figure S6C) all significantly diminishing the TNFα-induced increase in NF-κB reporter activity.

### 3.5. Elevated cAMP-Mediated Increased Nuclear Actin Monomer Reduced RelA/p65 Levels

**Figure 6:**
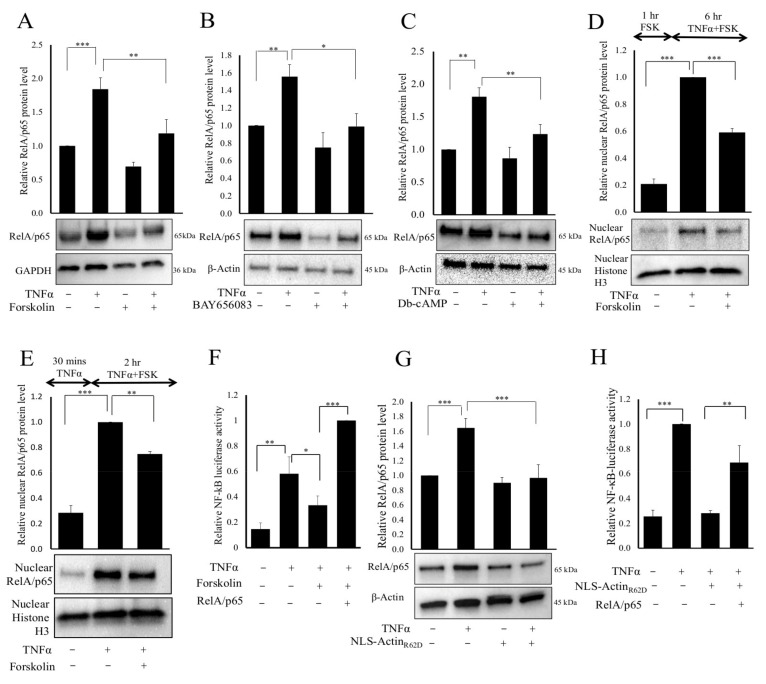
Cyclic-AMP-signalling reduces RelA/p65 protein levels. Serum starved VSMCs were stimulated with 25 µM forskolin ((**A**); *n* = 6), 5 µg/mL BAY60-6583 ((**B**); *n* = 6), or 200 µM Db-cAMP (**C**; *n* = 6) for 1 h before TNFα stimulation for a further 6 h. Total cell lysates were analysed for RelA/p65 and β-actin protein levels by Western blotting and densitometry analysis. Nuclear fractions were prepared and analysed for RelA/p65 protein levels ((**D**); *n* = 4). Cells were pre-treated with TNFα for 30 min prior to stimulation with forskolin for 2 h and nuclear fractions analysed for RelA/p65 levels ((**E**); *n* = 3). VSMCs were transfected with NF-κB-LUC and either a control expression plasmid or a plasmid overexpressing RelA/p65, as indicated. Serum-starved cells were treated with forskolin for 1 h before TNFα stimulation for 6 h. NF-κB reporter activity was quantified ((**F**); *n* = 4). Cells were infected with either Ad:Control or Ad:NLS-Actin_R62D_ and stimulated with TNFα for 6 h the following day. Total cell lysates were analysed for RelA/p65 and GAPDH protein levels by Western blotting and densitometry analysis ((**G**); *n* = 8). Cells were transfected with the NF-κB reporter and either a control or RelA/p65 expression plasmid and infected with either Ad:Control or Ad:NLS-Actin_R62D_ as indicated. Cells underwent 6 h stimulation with TNFα before NF-κB activity was measured ((**H**); *n* = 4). ANOVA with Student Newman Keul’s post hoc test. Data are mean ± SEM. *, ** and *** indicate *p* < 0.05, 0.01 and 0.001, respectively.

To investigate an alternative mechanism that might underlie cAMP and nuclear actin-mediated inhibition of NF-κB activity, we quantified the total cellular levels of RelA/p65 protein. Treatment with either forskolin (Figure 6A), BAY65-6083 (Figure 6B) or Di-butyryl-cAMP (Figure 6C) alone did not significantly affect RelA/65 levels. TNFα stimulation for 6 h significantly increased total cellular RelA/p65 protein levels (Figure 6A–C). However, treatment with forskolin (Figure 6A), BAY65-6083 (Figure 6B) or Di-butyryl-cAMP (Figure 6C) all significantly reduced the TNFα-stimulated RelA/p65 protein levels. Similar reductions in RelA/p65 protein levels were found in human VSMC following TNFα stimulation with forskolin co-treatment (Supplement Figure S7A). We next analysed nuclear fractions to test if cAMP signalling specifically reduced nuclear levels of RelA/p65 (Figure 6D). This analysis demonstrated significantly reduced levels of nuclear RelA/p65 in cells pre-treated with forskolin for 1 h before a 6-h stimulation with TNFα. To further test if this reduction was independent of changes in nuclear import of RelA/p65, we pre-treated cells with TNFα for 30 min to induce RelA/p65 nuclear translocation before treating cells with forskolin, whereupon nuclear RelA/p65 levels were also significantly reduced by forskolin (Figure 6E). Importantly, the forskolin-mediated inhibition of TNFα induced NF-κB-dependent reporter gene activity could be completely reversed by overexpression of exogenous RelA/p65 (Figure 6F), implying that reduced RelA/p65 levels are responsible for the observed cAMP-mediated inhibition of NF-κB activity. Since elevated cAMP signalling increases nuclear actin monomer levels [37], we tested whether increased nuclear actin monomer also reduced RelA/p65 protein levels. In control adenovirus infected cells (Ad:Control), TNFα stimulation significantly increased total RelA/p65 protein levels (Figure 6G). Basal levels of RelA/p65 were not significantly different in Ad:NLS-ActinR62D infected cells compared to controls. However, TNFα-stimulated RelA/p65 levels were significantly reduced in cells infected with Ad:NLS-Actin_R62D_. Following TNFα stimulation, an equivalent reduction in RelA/p65 protein levels was reported in human VSMC expressing NLS-Actin_R62D_ when compared to cells infected with a control adenovirus (Supplement Figure S7B). Furthermore, exogenous expression of RelA/p65 was able to partially rescue the inhibitory effects of NLS-Actin_R62D_ on TNFα-stimulated NF-κB reporter gene activity (Figure 6H).

**Figure 7:**
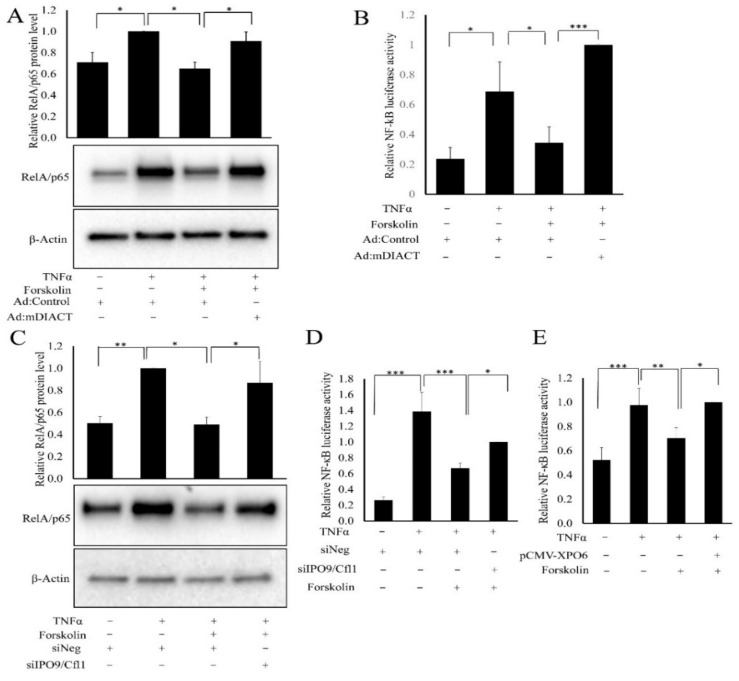
Reducing nuclear actin monomer levels rescues RelA/p65 protein levels and NF-κB activity in cells with elevated cAMP signalling. VSMCs were infected with either Ad:Control or Ad: mDIACT. The next day, cells were serum-starved and stimulated with TNFα and forskolin for 6 h, as indicated. Total cell lysates were analysed for RelA/p65 and β-actin protein levels by Western blotting and densitometry analysis ((**A**); *n* = 5). Cells were transfected with NF-κB-LUC and either a control or mDIACT expression plasmid ((**B**); *n* = 4). Serum-starved cells were stimulated with forskolin and TNFα for 6 h, as indicated, and luciferase activity quantified. VSMCs were transfected with either non-targeting siRNA (siNEG) or siRNA targeting IPO9 and CFL1 (siIPO9/Cfl1). Serum-starved cells were stimulated with forskolin and TNFα for 6 h, as indicated. Total cell lysates were analysed for RelA/p65 and β-actin protein levels by Western blotting and densitometry analysis ((**C**); *n* = 5). Cells were transfected with NF-κB-LUC and either siNEG or siIPO9/CFL1. Serum-starved cells were stimulated with forskolin and TNFα for 6 h, as indicated, and reporter activity measured ((**D**); *n* = 9). Cells were transfected with the NF-κB-LUC and either control or Exportin-6 (XPO6) expression plasmids. Serum-starved cells were stimulated with forskolin and TNFα for 6 h, as indicated, and luciferase activity quantified ((**E**); *n* = 6). ANOVA with Student Newman Keul’s post hoc test. Data are mean ± SEM. *, ** and *** indicate *p* < 0.05, 0.01 and 0.001, respectively.

We next used multiple approaches to antagonise cAMP-induced nuclear actin monomer levels, to directly test if the cAMP-induced reduction in RelA/p65 protein level and NF-κB activity is mediated by increased nuclear actin monomer levels. We initially overexpressed a nuclear localised active mutant of mDIA (mDIACT), which accumulates in the nucleus due to a cryptic nuclear localisation sequence (NLS), to specifically deplete nuclear actin monomer levels by inducing their polymerisation, as previously characterised [37,39]. Infection with Ad:mDIACT but not Ad:Control resulted in expression of mDIACT protein, detected using an anti-myc tag antibody (Supplement Figure S8). In control adenovirus infected cells, TNFα induced a significant increase in RelA/p65 levels, which was inhibited by forskolin (Figure 7A). However, this forskolin-mediated inhibition of RelA/p65 levels was completely reversed by expression of mDIACT (Figure 7A). Expression of mDIACT also completely reversed the forskolin-mediated inhibition of NF-κB reporter gene activity (Figure 7B). To further test the importance of cAMP-induced nuclear actin monomer, we silenced expression of importin9 (IPO9) and cofilin1 (CFL1), essential components of the complex responsible for import of actin monomers into the nucleus [41]. Transfection with siRNA targeting IPO9 and CFL1 resulted in a strong and significant reduction in the expression of IPO9 mRNA (Supplement Figure S9A) and CFL1 mRNA (Supplement Figure S9B) relative to control siNEG transfected cells. In cells transfected with control siRNA (siNEG), TNFα induced RelA/p65 protein levels, which were significantly reduced by forskolin (Figure 7C). Silencing of IPO9/CFL1 significantly increased RelA/p65 levels, reversing the forskolin-mediated inhibition (Figure 7C). Furthermore, IPO9/CFL1 silencing resulted in a significant but partial reversal of the forskolin inhibition of NF-κB reporter gene activity (Figure 7D). Lastly, we overexpressed exportin6 (XPO6), an essential component of the nuclear actin monomer export complex [42]. In control plasmid transfected cells, TNFα induced NF-κB reporter activity, which was inhibited by forskolin. Expression of XPO6 significantly increased reporter activity in forskolin-stimulated cells (Figure 7E). Taken together, these data demonstrate that cAMP-mediated reductions in RelA/p65 protein levels and NF-κB activity are mediated by increased nuclear actin levels.

### 3.6. Cyclic-AMP and Nuclear Actin Monomer Promote Proteasomal Degradation of RelA/p65 Protein

**Figure 8:**
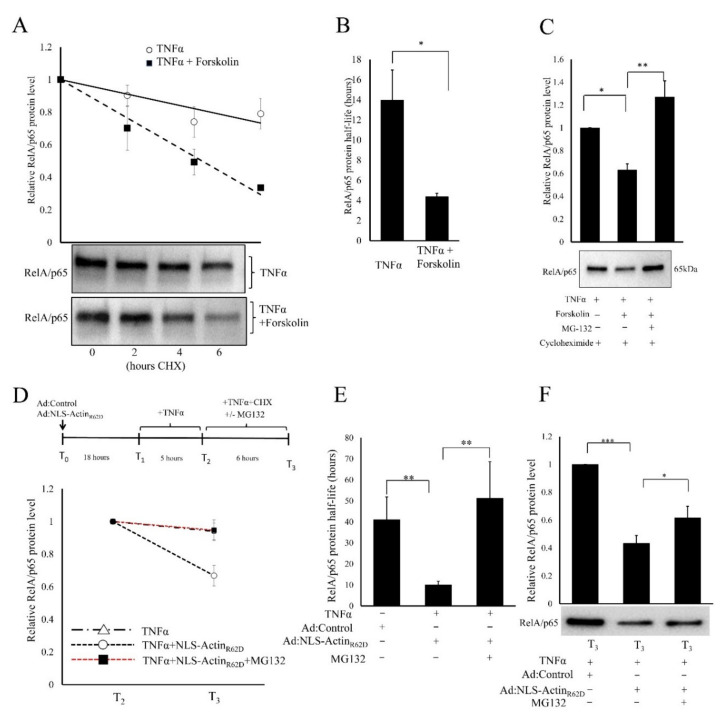
Cyclic-AMP and nuclear actin monomers induce RelA/p65 proteasomal degradation. Serum-starved VSMCs were stimulated with TNFα and forskolin for 5 h, as indicated. Following this, cells were incubated with cycloheximide for the indicated times. Total cell lysates were analysed for RelA/p65 protein levels by Western blotting and densitometry analysis ((**A**); *n* = 5). The RelA/p65 protein half-life was calculated ((**B**); *n* = 5). Serum-starved VSMCs were stimulated with TNFα and forskolin for 5 h, as indicated. Following this, cells were further treated with cycloheximide and MG-132 for a further 6 h. Total cell lysates were analysed for RelA/p65 protein levels by Western blotting and densitometry analysis ((**C**); *n* = 4). VSMCs were infected with either Ad:Control or Ad:NLS-Actin_R62D_ and stimulated with TNFα for 5 h before incubation with cycloheximide and MG132, as indicated. Total cell lysates were analysed for RelA/p65 protein levels by Western blotting and densitometry analysis, before calculating the degradation rate and half-life of RelA/p65 protein ((**D**–**F**); *n* = 5). ANOVA with Student Newman Keul’s post hoc test. Data are mean ± SEM. *, ** and *** indicate *p* < 0.05, 0.01 and 0.001, respectively.

We investigated if reduced stability of RelA/p65 protein was responsible for our observed reductions in RelA/p65 protein levels in response to elevated cAMP signalling and increased nuclear actin monomer. Cells were pre-treated with TNFα to induced NF-κB activation, followed by cycloheximide treatment to block new protein synthesis. RelA/p65 protein levels were then chased for 2, 4 and 6 h. This demonstrated that forskolin treatment increased the rate of turnover of RelA/p65 protein (Figure 8A). The half-life of RelA/p65 protein was significantly accelerated from 13.96 ± 3.00 h in TNFα treated cells to 4.37 ± 0.33 h in TNFα plus forskolin treated cells (Figure 8B). To test if this quickening of RelA/p65 half-life was dependent on proteasomal degradation, we treated cells with the proteasome inhibitor, MG132. These experiments were also performed in the presence of cycloheximide to eliminate any confounding effects of proteasome inhibition of IκBα levels, which may impact on NF-κB activation and cellular localisation. As described above, forskolin treatment increased the degradation of RelA/p65 (Figure 8C). However, this was completely prevented by co-treatment with MG132, implying involvement of proteasomal degradation in the cAMP-mediated reduction in RelA/p65 levels.

To test if the NLS-Actin_R62D_-mediated reduction in RelA/p65 protein level was due to increased proteasomal degradation, we measured the RelA/p65 protein half-life in cells treated with TNFα, TNFα plus NLS-Actin_R62D_ or TNFα plus NLS-Actin_R62D_ in the presence of the proteasome inhibitor, MG132 (Figure 8D–F). In TNFα-stimulated cells, the RelA/p65 half-life was 41.05 ± 10.84 h. This was higher than calculated in the previous experiment, presumably due to differences in serum concentration and effects of adenovirus infection. The RelA/p65 half-life was accelerated to 10.03 ± 1.73 h in cells expressing NLS-Actin_R62D_ (Figure 8E). Importantly, co-treatment with MG132 slowed the half-life up to 51.23 ± 17.47 h (Figure 8D,E) and reversed the NLS-Actin_R62D_ induced reduction in RelA/p65 protein levels (Figure 8F).

**Figure 9:**
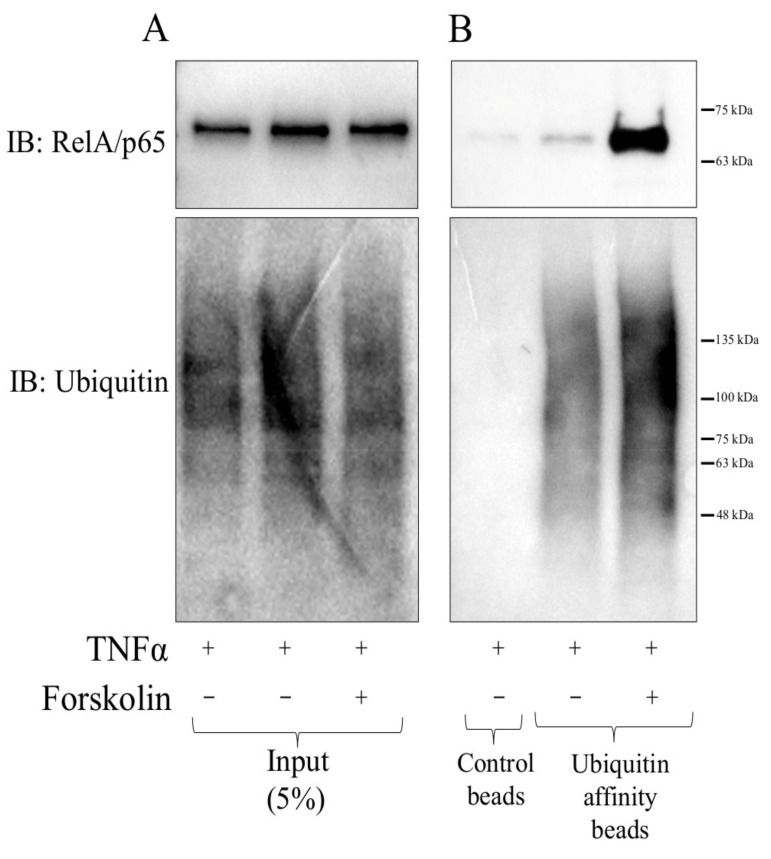
Cyclic-AMP signalling increases the ubiquitin-affinity purification of RelA/p65. Serum-starved VSMCs were stimulated with TNFα for 2 h before additional treatment with forskolin, cycloheximide and MG132 for a further 4 h. Total cell lysates (input) were analysed by Western blotting for RelA/p65 and ubiquitin (**A**). Ubiquitinated proteins were affinity purified from cell lysates using the Signal Seeker^TM^ ubiquitination assay kit and affinity purified RelA/p65 and ubiquitinated proteins analysed by Western blotting (**B**). Data are representative of two independent experiments.

Since proteasomal degradation is typically induced by protein ubiquitination, we tested if elevated cAMP signalling increases the association of RelA/p65 with ubiquitin using Signal-Seeker^TM^ ubiquitin affinity beads, that are coated with ubiquitin binding domain (UBD) of UBA1. Beads coated with a mutated UBD (CUB02), that does not bind ubiquitin, were used as a specificity control. Although input lysates (5% of sample used in Ub-affinity assay) contained equal levels of RelA/p65 protein (upper blot) and poly-ubiquitinated proteins (lower blot; Figure 9A), much more RelA/p65 was detected associated with ubiquitin-affinity beads incubated with lysates prepared from TNFα plus forskolin-stimulated cells compared to cells treated with TNFα only (Figure 9B). Densitometric analysis indicated that RelA/p65 band in the forskolin-stimulated Ub-affinity sample (Figure 9B, upper blot, lane 3) is approximately 3-fold bigger than the band in corresponding 5% input blot (Figure 9A, upper blot, lane 3). This indicates that approximately 15% of the total cellular RelA/p65 is ubiquitinated 4 h after forskolin stimulation.

## 4. Discussion

We present evidence that anti-inflammatory properties of cAMP signalling in VSMC are mediated, at least in part, via an increase in nuclear actin monomer levels, which promote proteasomal degradation of the NF-κB subunit RelA/p65.

Our first clue came from analysis of RNA-seq transcriptomic data of cells stimulated with forskolin and cells overexpressing a nuclear targeted polymerisation defective actin mutant (NLS-Actin_R62D_). These data highlighted enrichment of several gene ontology terms associated with inflammation and regulation of NF-κB. Additionally, analysis of the promoter regions of genes identified by RNAseq as being repressed by cAMP elevation or NLS-Actin_R62D_ identified a significant enrichment of NF-κB binding elements, suggesting that NF-κB-dependent inflammatory genes might be sensitive to cAMP-mediated increases in nuclear actin levels. We went on to demonstrate that elevation of cAMP levels using the adenylate cycle agonist forskolin, cAMP analogues such as dibutyryl-cAMP or synthetic agonists of the adenosine A2B-receptor or directly increasing nuclear actin monomer levels by expressing NLS-Actin_R62D_ all inhibit basal and TNFα-stimulated NF-κB activity and expression of NF-κB target genes. Mechanistically, inhibition of NF-κB transcriptional activity and inflammatory gene expression was associated with a reduction in the total cellular and nuclear levels of RelA/p65 rather than a change in its cytoplasmic:nuclear ratio. Indeed, nuclear RelA/p65 levels were reduced in response to forskolin stimulation even after RelA/p65 nuclear translocation was induced by pre-treatment with TNFα, consistent with them being independent of changes in nuclear:cytoplasmic translocation. We found instead that both cAMP signalling and nuclear actin monomer elevation accelerated the turnover of RelA/p65, and inhibition with MG132 showed this to be proteosome-mediated. Furthermore, we showed that RelA/p65 became ubiquitinated after forskolin treatment of VSMCs. Moreover, the reductions in RelA/p65 levels were functionally significant because preventing cAMP-induced increases in nuclear actin monomer levels by promoting its nuclear polymerisation using a nuclear localised active mutant of mDIA, completely rescued RelA/p65 levels and NF-κB activity. Likewise, preventing nuclear import of actin monomers by silencing components of the import complex (i.e., IPO9 and CFL1) also completely rescued RelA/p65 levels and partially rescued NF-κB activity. Finally, enhancing nuclear export of actin monomer by overexpressing XPO6 also rescued NF-κB activity in forskolin-stimulated cells.

Proteasomal degradation of NF-κB subunits, including RelA/p65 and cRel/p50, has previously been implicated in regulation of NF-κB activity [43,44,45], with RelA/p65 degradation being linked to termination of NF-κB-dependent transcription [46,47]. Interestingly, RelA/p65 ubiquitination occurs predominantly within the nucleus and has been reported to be dependent on DNA binding. For example, ubiquitination and proteasomal degradation of promoter bound RelA/p65 ensures transient transcriptional responses after TNFα stimulation [47].

The ability of cAMP and cAMP modulating agents to regulate inflammatory responses has been recognised for several decades [48,49]. In 1974, Bourne et al. first proposed “*general inhibitory action of cAMP on immunological and inflammatory functions of leucocytes”* [49]. Since then, numerous studies have documented anti-inflammatory properties of cAMP signalling in a wide variety of cell types [23,50,51,52], although some also reported pro-inflammatory effects [53,54]. This divergence may arise due to cell type specific effects, or differences in the nature of the pro-inflammatory stimulus or timepoint studied. However, most studies describe anti-inflammatory properties of cAMP signalling, and a variety of underlying mechanisms have been proposed, many focussed on the canonical pro-inflammatory transcription factor, NF-κB. PKA-mediated inhibition of IκBα degradation [50,55], inhibition of NF-κB DNA binding [24,56] or changes in NF-κB dimer composition [57] have all been implicated. Our results pointed instead to a novel mechanism mediated by increased nuclear actin monomer levels, which induce proteasomal degradation of RelA/p65.

Remodelling of the actin cytoskeleton has previously been implicated in mediating some of the effects of cAMP in VSMCs, including regulation of cell proliferation and migration [34,37,58], but not until now has it been linked to inflammation. Elevation of cAMP inhibits RhoGTPases [30], which reduces cytoplasmic actin polymerisation and actin stress fibre formation, resulting in inhibition of actin dependent transcriptional co-factors such as MRTF-A/B [34] and YAP/TAZ [33] and the activity of their respective transcription factor partners, SRF and TEAD. Interestingly, all these inhibitory effects of cAMP are mediated via an increase in the levels of actin monomer within the nucleus, which occurs secondary to cAMP-induced changes in the cytoplasmic actin cytoskeleton in multiple cell types, including vascular smooth muscle [37] cells, cardiac fibroblasts [59] and astrocytes [60].

Nuclear actin was originally identified in the 1960s [61], and has become widely recognised recently as an important factor in chromatin remodelling, cell differentiation and transcription of certain genes [62]. Our novel findings demonstrate an additional role in protein degradation. Precisely how this occurs is currently unknown, but our data implicate ubiquitin-mediated proteasomal degradation.

Further evidence for an association between actin and inflammation comes from genetic studies. Indeed, several immune-mediated diseases are associated with genes involved in actin cytoskeleton remodelling [63,64]. For example, the first so called ‘actinopathy’, Wiskott–Aldrich syndrome (WAS) is characterised by immunodeficiency caused by loss-of-function mutations in the WAS protein (WASp), which regulates actin polymerisation [58,65,66].

Whether nuclear actin-mediated RelA/p65 degradation occurs only in VSMCs is not yet known. Whether it applies in other cells, such as cardiac fibroblasts, that show similar cytoskeletal changes in response to cAMP will require further investigation.

The precise mechanisms by which nuclear actin promotes RelA/p65 degradation also require future studies beyond the present scope. Using ubiquitin-affinity pull-down assays, we demonstrated increased association of RelA/p65 with ubiquitin-affinity beads in cells treated with forskolin. Our data indicate that approximately 15% of the cellular RelA/p65 is ubiquitinated following forskolin stimulation. However, these assays did not detect multiple bands of increasing molecular weight, as expected if RelA/p65 was poly-ubiquitinated. This may reflect mono- rather than poly-ubiquitination of RelA/p65 in our experiments. RelA/p65 mono-ubiquitination has previously been implicated in the regulation of NF-κB activity [67], although this form of ubiquitination is not associated with increased proteasomal degradation. However, as our assays were performed under non-denaturing conditions, we cannot exclude the possibility that cAMP signalling enhances ubiquitination of another protein component of RelA/p65 containing complexes and this triggers the destruction of the associated RelA/p65. Nevertheless, our data clearly demonstrate that cAMP-signalling and increased nuclear actin levels induce the proteasomal degradation of RelA/p56 leading to attenuated NF-κB activity.

This study highlights for the first time the important role of nuclear actin monomer in mediating at least some of the anti-inflammatory properties of cAMP. We believe that ours is the first evidence that it can promote accelerated RelA/p65 degradation and inhibit inflammatory gene expression. Defining the mechanism by which nuclear actin promotes RelA/p65 is clearly now an important future goal. However, the implication of our data is that dysregulation of nuclear actin levels may represent an important mechanism that primes a pro-inflammatory phenotype during development of cardiovascular disease. Moreover, targeting nuclear actin dynamics may be a new strategy to combat cardiovascular diseases including atherosclerosis and angioplasty restenosis and other diseases associated with chronic or excessive inflammation.

## Supplementary Materials

The following supporting information can be downloaded at: https://www.mdpi.com/article/10.3390/cells11091414/s1, Figure S1: Table of qPCR primers; Figure S2: Forskolin stimulation increases nuclear actin monomer levels.Figure S3: Adenoviral mediated expression of FLAG-tagged NLS-ActinR62D.;.Figure S4: Cyclic-AMP signalling inhibits NF-κB-dependent transcription in human VSMC; Figure S5: Cyclic-AMP and nuclear actin monomers do not affect RelA/p65 nuclear translocation; Figure S6: Cyclic-AMP signalling and nuclear actin monomer inhibit NF-κB activity in human VSMC.; Figure S7: Forskolin and nuclear actin monomer reduced RelA/p65 protein levels in human VSMC; Figure S8: Adenovirus-mediated expression of MYC-tagged mDIA-CT; Figure S9: Efficiency of IPO9 and CFL1 silencing.
