# Supplementary material for: Cyclic-AMP Increases Nuclear Actin Monomer Which Promotes Proteasomal Degradation of RelA/p65 Leading to Anti-Inflammatory Effects
Source: Cells. 2022 Apr 21;11(9):1414. doi: 10.3390/cells11091414 (PMC9101168; doi:10.3390/cells11091414)
Supplement: Supplementary file 1 [file cells-11-01414-s001.zip › cells-1581959-supplementary.pdf]

SUPPLEMENT FIGURES

GENE	FORWARD PRIMER (5'-3')	REVERSE PRIMER (5'-3')
VCAM1	TCCGTTGTTCTGACATGTGCTGCT	CGTCAGTGTGGATGTAGCCCCTTC
ICAM1	CACAGCTCTCAGTAGTGCTGCTCC	AGCATGAGAAATTGGCTCCGTGGT
HDAC9	GCGGTGGCAAGCACAGAGGTTA	GGGGTGGAGAGCTTTGATCCAGTG
TLR5	TCTCTCCGGGCAGTTTACCTGCTA	TGAGTTATGTCCAAGGCACGGAGC
F11R	TCATCCAGTGGCATCACCTTCAGC	ATGCTGACCTCCCCGTAGTTCTGA
IL1RL2	ATGGCTTTCCTACTTCTGGCCGTG	CAGCTTCTCGTCATCTGGAGCCTG
CD180	GTGGTTCCCAGCATTTCGGCTCTA	AGCTAACGCTCAGAGCCAAGCAAT
CXCL10	AGAGCTGGTCCGAATCTTCCCTCA	GAGCTAGGGAGAAGGAGGGTGTGT
GATA3	CACTCCAGTCCGCATCTCTTCACC	ACTCTTTCTCATCTTGCCTGGCCG
36B4	AGCCAAGGTCGAAGCAAAGGAAGA	GACTTGGTGTGAGGGGCTTAGTCG
CXCL2	CAGACGCAGTCAGAGGGCTTTCAA	ACAGAAATAACAGTCGTCCCGCCC
IL6	GAGCCCACCAGGAACGAAAGTCAA	CCGGACTTGTGAAGTAGGGAAGGC
MCP1	ATGCAGGTCTCTGTCACGCTTCTG	TGCCAGTGAATGAGTAGCAGCAGG
NFKB1A	GTCAGAATTCACGGAAGATGAGTTGC	AGTCCACGTTCCTTTGGCCACTTT
IL1B	GAAGCAGCTATGGCAACTGTCCCT	GCTCTCATCTGGACAGCCCAAGTC

Supplementary Figure S1: Table of qPCR primers

Quantitative RT-qPCR primers were designed using NCBI Primer designing tool and used at a final concentration of 0.5 μM

Supplementary Figure S2

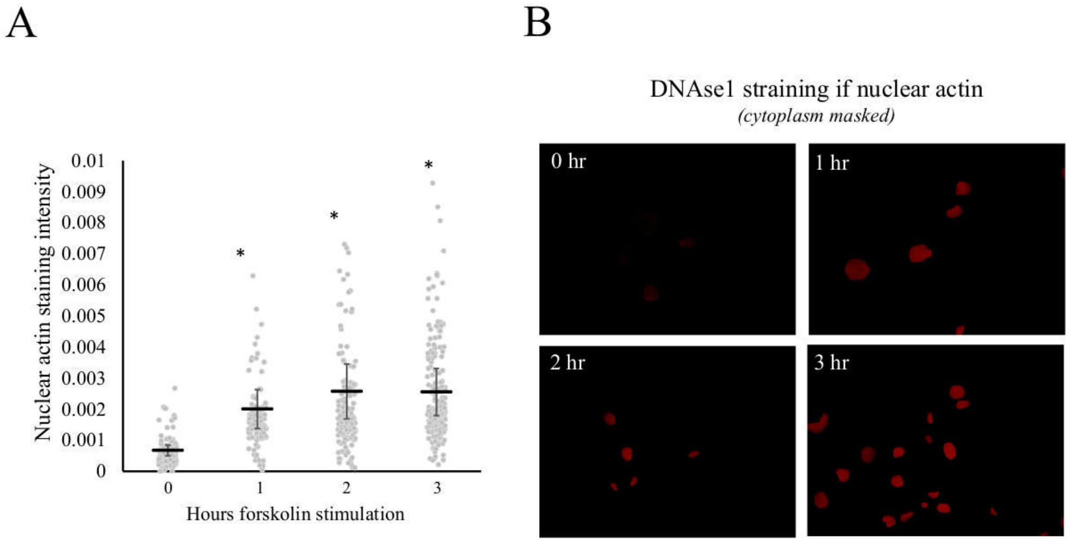

Supplementary Figure S2: Forskolin stimulation increases nuclear actin monomer levels

VSMC were serum deprived for 24 hours before stimulation with 25 µM forskolin for the indicated times. Cells were fixed and stained with Alexa-Fluor-594 conjugated DNAse1 to detect actin monomers. Nuclei were counterstained with Hoechst 33342 and the resulting Hoechst-stained images used to create a cytoplasmic mask. Nuclear intensity of the Alexa Fluor 594 DNAse1 was quantified using Cell Profiler software. (A) Quantification of nuclear actin monomer. Grey dots indicate individual data points from single nuclei. Bars represent mean intensity from three independent experiments. (B) Representative micrographs of Alexa Fluor-594 stained cells with cytoplasmic mask applied. One was ANOVA with Student Newman Kuels post-test; * indicates $p<0.05$.

Supplement Figure S3

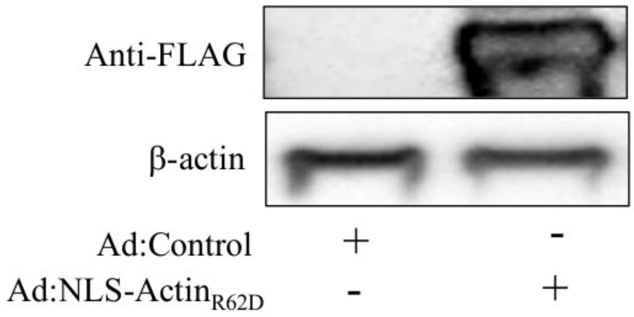

Supplementary Figure S3: Adenoviral mediated expression of FLAG-tagged NLS-Actin$_{R62D}$

VSMC were infected with either Ad:Control or Ad:NLS-Actin$_{R62D}$. The next day total cell lysates were analysed by western blotting for expression of FLAG-tagged NLS-Actin$_{R62D}$ and total cellular levels of β-actin.

Supplement Figure S4

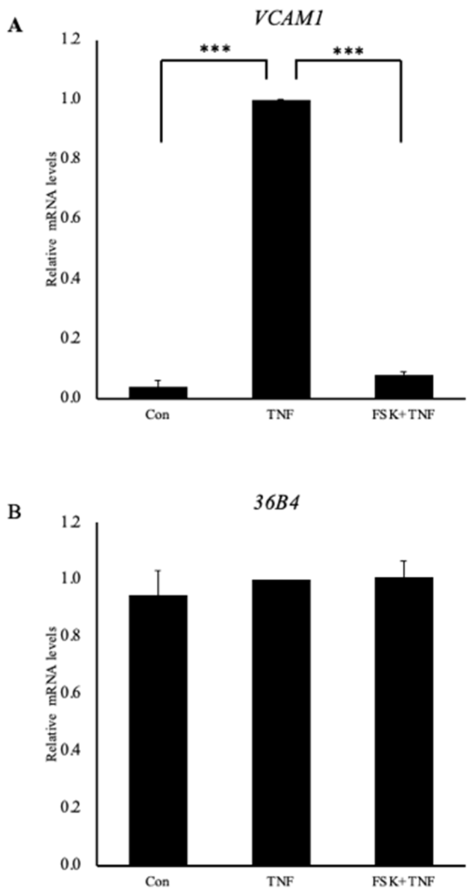

Supplementary Figure S4: Cyclic-AMP signalling inhibits NF-κB-dependent transcription in human VSMC.

Human VSMC were serum starved for 24 hours before 1 hour stimulation with forskolin (100μM) and subsequent TNFα (50ng/ml) stimulation for 8 hours. Total RNA was extracted and analysed for total mRNA levels of VCAM1 and housekeeping gene 36B4 using RT-qPCR (A, B; n=3).

Supplementary Figure S5

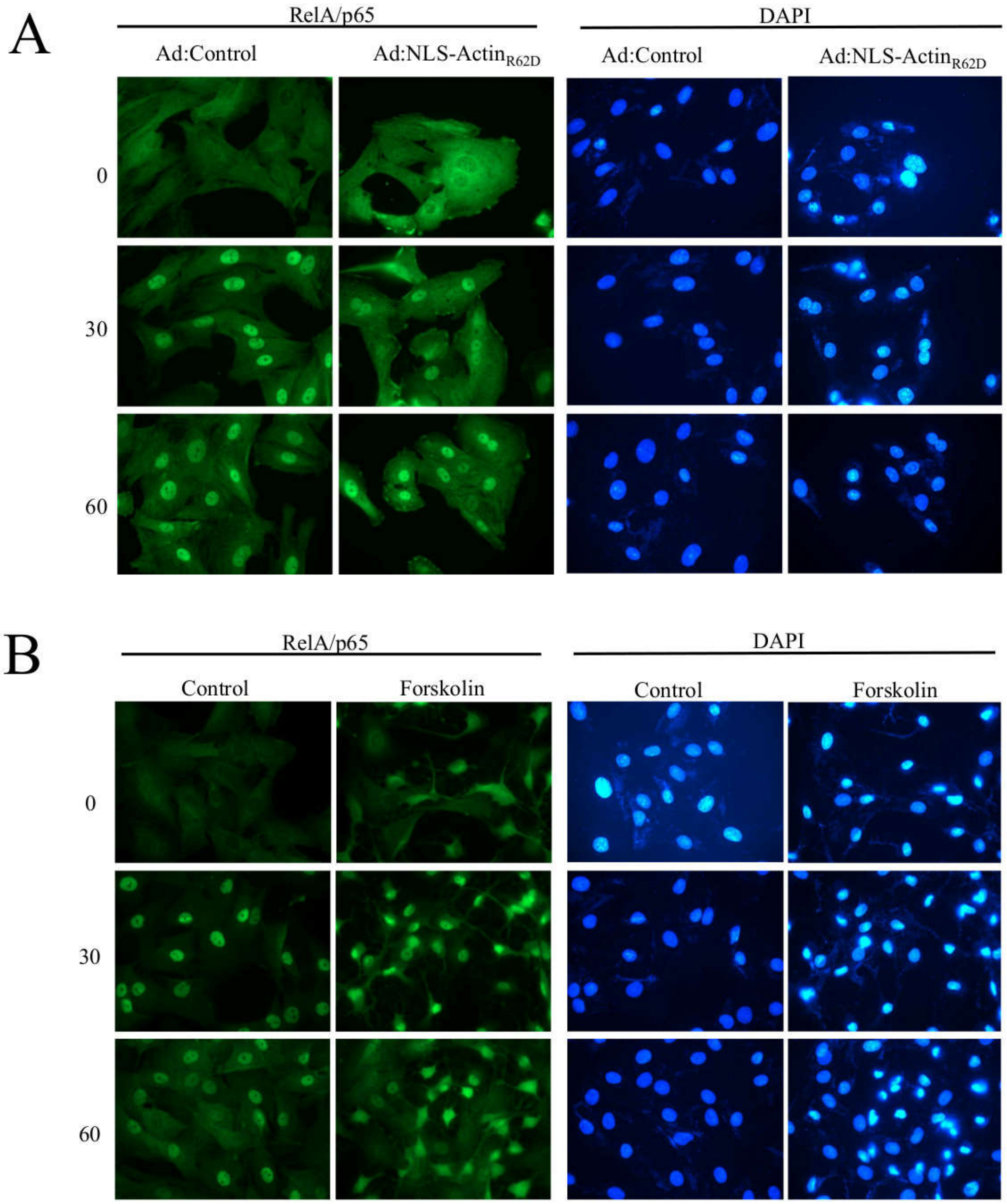

Supplementary Figure S5: Cyclic-AMP and nuclear actin monomers do not affect RelA/p65 nuclear translocation

Cells were infected with control adenovirus (Ad:Control) or virus expressing NLS-Actin$_{R62D}$ (Ad:NLS-Actin$_{R62D}$) 24 hours before TNFα stimulation (A). Serum starved VSMCs were pre-treated with 25 μM forskolin for 1 hour prior to TNFα stimulation for the indicated times (B). Cells were fixed and stained for RelA/p65 and nuclei counter stained with Hoechst 33342.

Supplementary Figure S6

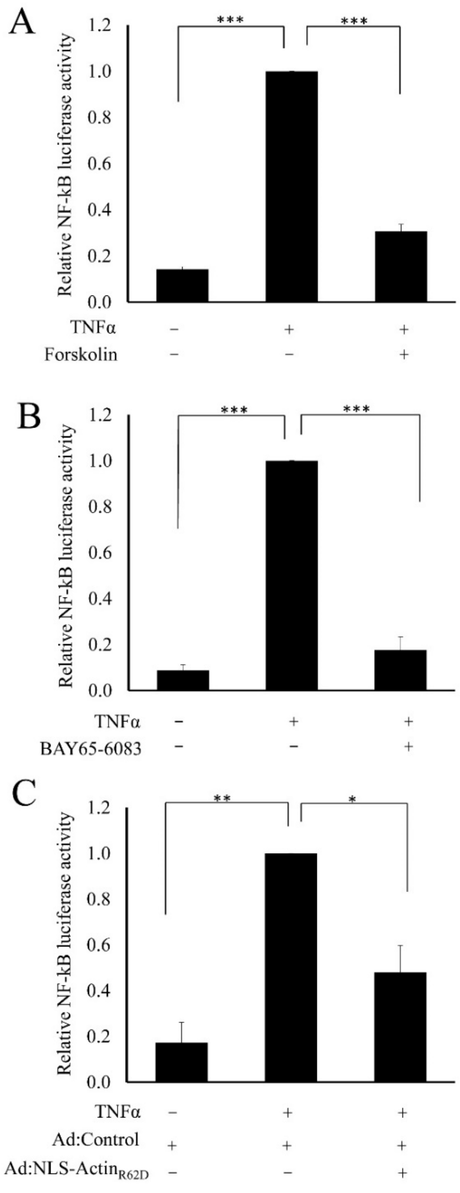

Supplementary Figure S6: Cyclic-AMP signalling and nuclear actin monomer inhibit NF-κB activity in human VSMC.

Human VSMC were transfected with NF-κB-LUC luciferase reporter plasmid. The following day, cells were (A; n=3) pre-treated with forskolin for 1 hour before a 6 hour stimulation with TNFα, (B; n=4) pre-treated with BAY65-6083 for 1 hour before a 6 hour stimulation with TNFα, (C; n=4) infected with Ad:Control or Ad:NLS-Actin$_{R62D}$ and then stimulated with TNFα for 6 hours the day after adenovirus infection.

Supplementary Figure S7

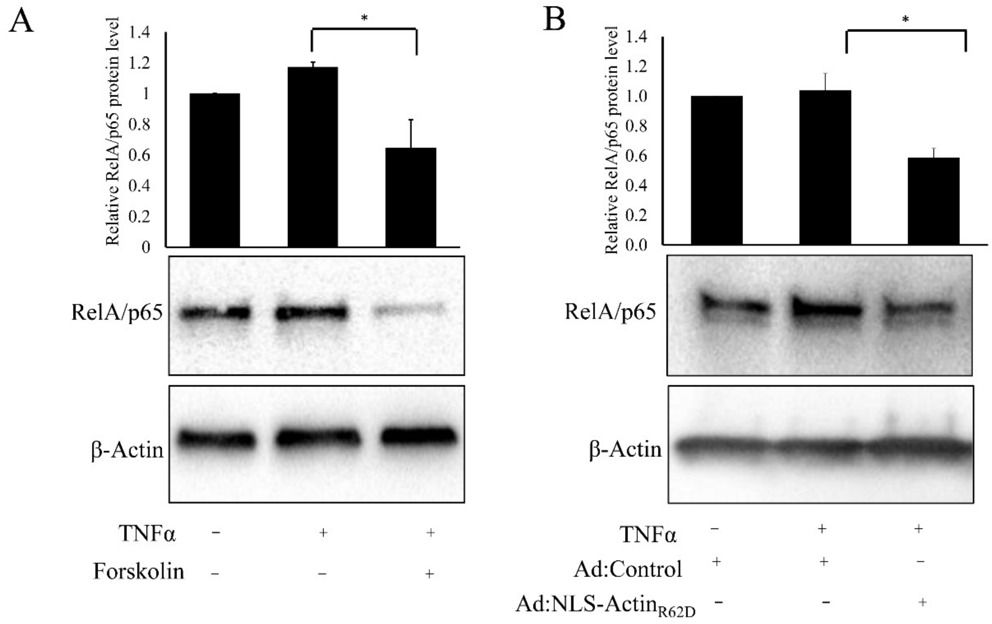

Supplementary Figure S7: Forskolin and nuclear actin monomer reduced RelA/p65 protein levels in human VSMC

Human VSMC were serum starved for 24 hours before stimulation with forskolin and TNFα as indicated for 6 hours (A; n=3). Cells were infected with either Ad:Control or Ad:NLS-Actin$_{R62D}$. The next day, cells were stimulated with TNFα for *** hours (B; n=3). Total cell lysates were analysed for RelA/p65 and β-actin levels by western blotting.

Supplementary Figure S8

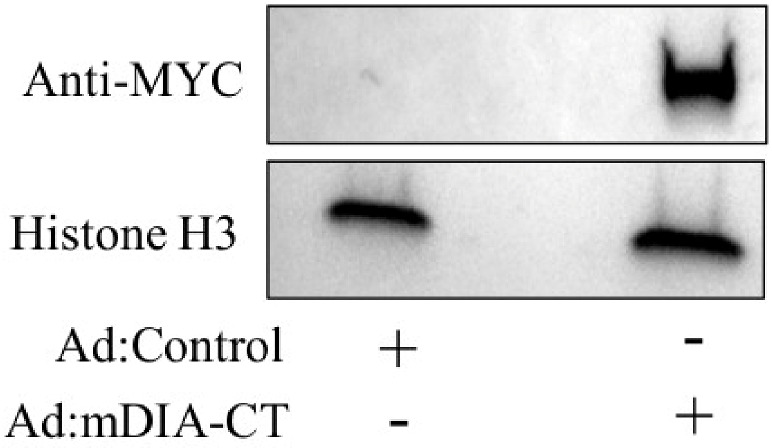

Supplementary Figure S8: Adenovirus-mediated expression of MYC-tagged mDIA-CT

Cells were infected with either Ad:Control or Ad:mDIACT. The next day total cell lysates were prepared an analysed by western blotting using anti-MYC tag antibody and Histone H3.

Supplementary Figure S9

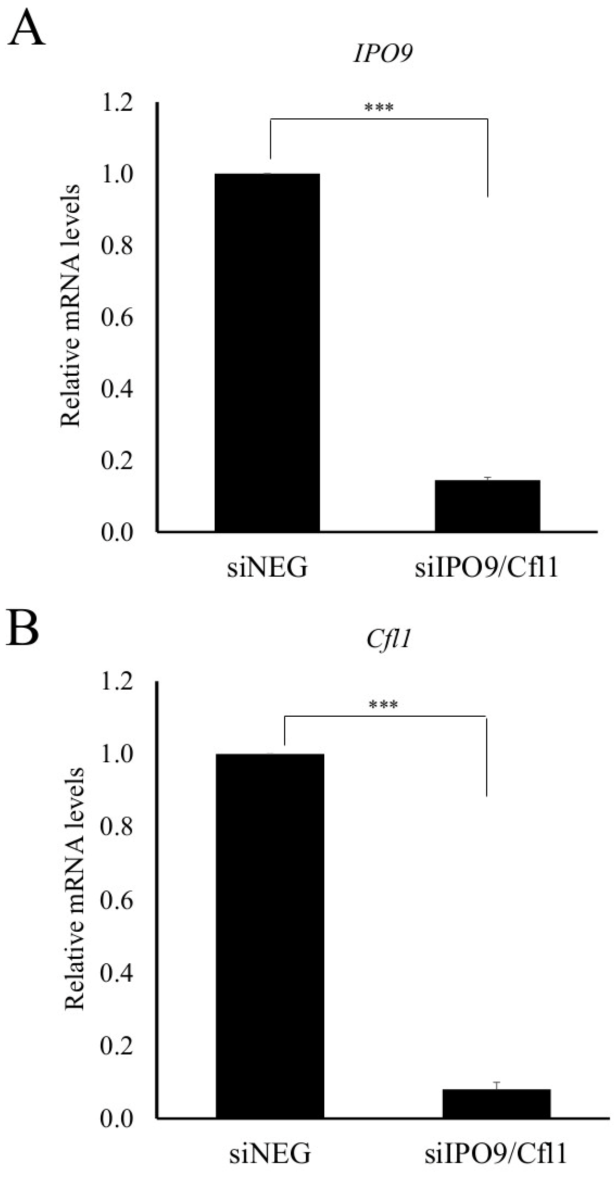

Supplementary Figure S9: Efficiency of IPO9 and CFL1 silencing

Cells were transfected with siRNA targeting IPO9 and CFL1. The next day, total RNA was prepared and analysed by RT-qPCR for mRNA levels of IPO9 (A) and CLF1 (B). Paired student t-test. *** indicated $p<0.001$.
